# Normobaric oxygen may attenuate the headache in patients with patent foramen povale and migraine

**DOI:** 10.1186/s12883-023-03059-z

**Published:** 2023-01-27

**Authors:** Mengqi Wang, Duo Lan, Chaitu Dandu, Yuchuan Ding, Xunming Ji, Ran Meng

**Affiliations:** 1 Department of Neurology, Xuanwu Hospital, Capital Medical University, Beijing, 100053 China; 2 Advanced Center of Stroke, Beijing Institute for Brain Disorders, Beijing, 100053 China; 3 National Center for Neurological Disorders, Xuanwu Hospital, Capital Medical University, Beijing, 100053 China; 4 Department of Neurosurgery, Wayne State University School of Medicine, Detroit, MI 48201 USA

**Keywords:** Patent foramen ovale, Migraine, Normobaric oxygenation, Hyoxemia, Blood gas analysis

## Abstract

**Background and purposes:**

There has been both great interest in and skepticism about the strategies for headache inhibition in patients with patent foramen ovale and migraines (PFO-migraine). Furthermore, many questions remain about the fundamental pathophysiology of PFO-migraines. Herein, the inhibiting effect of normobaric oxygenation (NBO) on PFO-migraine was analyzed.

**Methods:**

This real-world self-control study consecutively enrolled patients during the ictal phase of migraines who had patent foramen ovale (PFO) confirmed by Trans esophageal Ultrasound(TEE). After comparing the baseline arterial oxygen partial pressure (PaO_2_) in their blood gas with that of healthy volunteers, all the patients with PFO-migraine underwent treatment with NBO (8 L/min. for 1 h/q8h) inhalation through a mask. Their clinical symptoms, blood gas, and electroencephalograph (EEG) prior to and post-NBO were compared.

**Results:**

A total of 39 cases with PFO-migraine (in which 36% of participants only had a small-aperture of PFO) and 20 non-PFO volunteers entered the final analysis. Baseline blood gas analysis results showed that the PaO_2_ in patients with PFO-migraine were noticeably lower than PaO_2_ levels in non-PFO volunteers. After all patients with PFO-migraines underwent NBO treatment, 29(74.4%) of them demonstrated dramatic headache attenuation and a remarkable increase in their arterial PaO_2_ levels after one time treatment of NBO inhalation (*p* < 0.01). The arterial PaO_2_ levels in these patients gradually went down during the following 4 h after treatment. 5 patients finished their EEG scans prior to and post-NBO, and 4(80%) were found to have more abnormal slow waves in their baseline EEG maps. In the follow up EEG maps post-NBO treatment for these same 4 patients, the abnormal slow waves disappeared remarkably.

**Conclusions:**

Patients with PFO–migraine may derive benefit from NBO treatment. PFOs result in arterial hypoxemia due to mixing of venous blood, which ultimately results in brain hypoxia and migraines. This series of events may be the key pathologic link explaining how PFOs lead to migraines. NBO use may attenuate the headaches from migraines by correcting the hypoxemia.

## Highlights

Brain hypoxia may be the key point of pathological link between PFO and migraine.

NBO may be a feasible promising method on PFO–migraine control.

The size of PFO aperture may be not very matched with the severity and the incidence of migraine.

## Introduction

It is well known that patent foramen ovale (PFO), a congenital right-to-left interatrial shunt, is one of the main etiologies of migraines. A prior study revealed that the degree of leak via the PFO tunnel may impact the frequency and intensity of migraines [1]. The pathogenesis of migraine with PFO (PFO-migraine) is hypothesized to entail paradoxical embolism, cortical spreading depression (CSD), and vasospasm, all of which lead to decreased oxygen content [2–5]. Therefore, based on the aforementioned mechanisms current standard treatments for blocking PFO- migraine during the ictal phase mainly comprise of medications and PFO closure [6, 7]. Several studies have discovered a reduction in migraine frequency and duration following PFO closure. However, it is still unknown how to reduce the severity of a migraine during the ictal phase [8, 9]. In addition, not all patients with PFO are candidates for PFO closure. Currently, guidelines recommend that only patients with intractable migraines and moderate to severe right-to-left shunt (RLS) detected by TEE or bubble-TCD undergo PFO closure [10]. However, excluded from this criteria are patients with mild RLS who also complain of severe migraine. As such, in addition to the above-mentioned therapies, more consideration should be given to alternative migraine-inhibiting treatments. In this paper, we aim to explore alternative migrained-inhibiting treatments by conducting a real-world self-control study exploring the use of normobaric oxygenation (NBO) as a novel therapy for PFO-migraine treatment.

## Method

### Study population and clinical features

Consecutive patients confirmed with both PFOs and migraines were prospectively recruited into this real-world self-control study from January 2021 through October 2021 in Xuanwu Hospital, Capital Medical University, China after signing an informed consent form. This study was conducted in conformity with the Helsinki Declaration of 1964 and its revisions.

Inclusion criteria: patients with migraine, ages from 18 to 80 years, with transoesophageal echocardiography (TEE) and/or contrast-enhanced transcranial Doppler (bubble-TCD) confirmed PFO, no gender preference. Exclusion criteria included subjects with small-vessel disease, atrial fibrillation or other cardiogenic diseases, history of stroke caused by vascular stenosis and hypercoagulable status, and migraine that can be explained by causes other than PFOs. The medical record was thoroughly examined for demographic information and clinical characteristics.

### PFO assessment

Both bubble-TCD and TEE were used to confirm the PFO diagnosis. TEE was used to measure the length of the PFO tunnel and to examine patients who were deemed inappropriate for bubble-TCD due to factors such as a poor temporal bone window. An expert neuroscientist carried out and evaluated the bubble-TCD and TEE studies. According to bubble-TCD, the size of PFO was classified as small (1–9 bubbles), medium (10–20 bubbles), or large (> 20 bubbles) [11].

### Migraine assessment

To quantify the severity of migraines, we employed migraine frequency (number of attacks separated by at least 48 h of pain-free intervals each week), severity (0–10 ratings; 0: no pain, 10: worst agony conceivable), triggering factors, and presence or absence of aura as clinical measures for migraine. Patients recorded this baseline data during the last ictal phase before 7-day NBO treatment. Additionally, during ictal phase of migraine, patients recorded severity before and after 1-hour NBO treatment respectively.

### Normobaric oxygenation (NBO) strategy

**Fig. 1:**
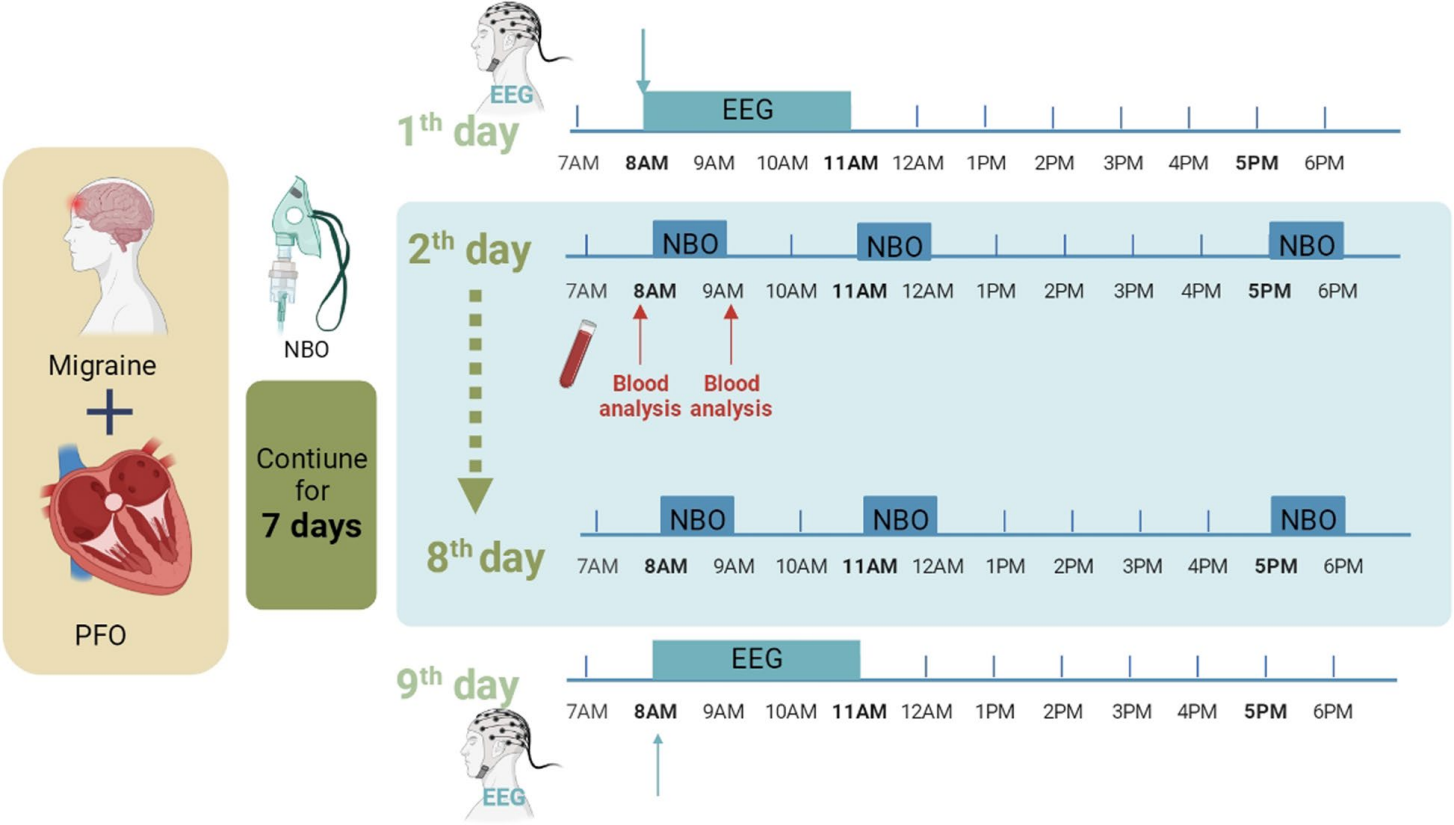
Flowchart of the study. PFO: patent forman ovale; NBO: normobaric oxygenation; EEG: electroencephalo-graph

In this study, patients with PFO-migraines in the ictal phase received NBO therapy by inhaling 8 L/min of oxygen using a face mask for 1 h, three times a day (8AM, 11AM and 5PM) [12]. All patients continuously underwent 7 days of NBO treatment, and did not take any painkillers nor any other drugs that might lessen migraine intensity with or without PFO simultaneously. During this period, the intensities of migraine onsets were recorded in a diary. In addition, blood gas measurements from the arterial and venous systems were evaluated before and after treatment (Fig. 1).

### Comparison of oxygen partial pressure

While calm, healthy volunteers underwent blood gas analysis and patients with PFO-migraine prior to NBO inhalation underwent blood gas analysis. Oxygen partial pressure (PaO_2_), carbon dioxide partial pressure (PaCO_2_), and oxygen saturation (SpO) were measured and compared between PFO-migraine patients and the healthy volunteers. After 1-hour of NBO treatment, follow up blood gas analysis was done in the patients with PFO-migraine. The data of arterial and venous PaO_2_, PaCO_2_, and SpO at baseline and post-NBO treatment were compared. In addition, to document PaO_2_ dynamic fluctuation, arterial blood gas analysis in the patients with PFO-migraine was taken at four times points: at baseline pre-NBO, the instant post-NBO, the second hour post-NBO, and the fourth hour post-NBO, respectively (Fig. 1).

### Brain electrophysiological assessment

On the day before and the day following the end of the 7-day NBO therapy, patients with migraine-PFO completed a 4-hour Electroencephalograph (EEG) scan in the morning (8 AM) in a warm, quiet room while remaining calm. Two neurologists independently assessed the data to see how NBO affected brain activity (Fig. 1).

### Statistical analysis

All data were normally distributed and confirmed by the Shapiro Wilk test. Descriptive statistics were calculated for all data. Group comparisons for continuous variables were analyzed using the Student’s t-test or Wilcoxon rank sum test. Analysis of variance was used to calculate the differences in mean between the two samples. Categorical variables were reported as numbers and analyzed by Chi-square test or Fisher exact test. Two-sided *p*-values of < 0.05 were considered statistically significant. All statistical analyses were performed using the SPSS version 21.0 for Windows.

## Results

### Demographic and clinical characteristics

A total of 39 patients confirmed as PFO and migraine [15 males and 24 females, mean age 44.11 (19–74) years] and 20 healthy volunteers [14 males and 6 females, mean age 58.625 (35–72) years] were enrolled. PFO-migraine patients and healthy volunteers were matched according to age and gender. The average VAS score, used to assess the severity of migraines, was 4.62 (2 to 7); further details are shown in Table 1. Other vague symptoms including dizziness, paroxysmal face numbness and limb weakness, chest tightness, and palpitations were reported by some individuals. The average aperture of PFO was 2.13 ± 0.64 mm, and TEE detected a variety of leak severities through the PFO holes. Additionally, there was no correlation between the incidence of the migraine and the PFO’s aperture (*p* = 0.787). 35 patients out of 39 (89.7%) underwent NBO therapy; 4 patients discontinued NBO after receiving the first session of therapy, as they could not tolerate to the facemask-related nausea and discomfort.

**Table 1:** The characters of patients with migraine and PFO

Parameter（*n*=39）	Results
Site of the headache	
Frontal brain	5
Parietal part	8
Occipitoparietal part	3
Temporal part	4
Whole brain	14
One side of hemisphere	2
Uncertain site	4
Frequency of the headache	
0-5times/month	22
5-10times/month	11
>10 times/month	6
Aura	
With aura	25
Without aura	14
Intensity of the headache	
VAS	4.62 (2-7)

### Baseline arterial blood gas evaluation

Prior to NBO treatment, baseline PaO_2_ and PaCO_2_ from arterial blood gasses in 35 patients with migraine-PFO were compared to those in 20 healthy volunteers who had not been given oxygen. Baseline PaO_2_ levels in healthy volunteers were substantially higher than those in PFO-migraine patients (89.24 ± 1.69 vs. 84.14 ± 3.25, *p* = 0.04). In contrast, there was no statistically significant difference in the baseline PaCO2 levels between the two groups (41.57 ± 1.01 vs. 31.8 ± 1.63 *p* = 0.338).

### Migraine relief after NBO

During the inpatient period, 35 PFO-migraine patients in the ictal phase underwent 7-days of continuous NBO treatment (8 L/min for 1 h, three times daily). 30 of the 35 patients (85.7%) reported that the frequency or intensity of the migraine attenuated after NBO, including patients with a small aperture PFO (25.8%); only 4(11.4%) patients complained that the severity of their migraines had not decreased after NBO. Prior to NBO treatment, the average VAS was 5.23 (7 to 3), after 7-days of NBO use, the average VAS was 1.59 (4 to 0) (*p* < 0.01), NBO treatment attenuated the intensity of migraines (*p* = 0.024, Tables 2).

**Table 2:** Data of blood gas in both artery and venous prior to versus post-NBO

	Pre- NBO	Post- NBO	*P*-value
VAS	5.23(7 to 3)	1.59(4 to 0)	<0.01
PaO_2_			
Arterial	84.14(34.9-103)	108.08(72-189)	<0.01
Venous	48.52(25.3-71.5)	51.85(25.6-118)	0.492
PaCO_2_			
Arterial	41.57(36.5-59.8)	40.22(35.7-51.4)	0.074
Venous	46.23(35.257.8)	46.6(38-62.4)	0.739
SpO			
Arterial	95.35(63.3%-99.3%)	97.53(95.3-99.5)	0.111
Venous	76.29(41.3-95.1)	78.18(42.1-98.7)	0.590
TCO_2_			
Arterial	21.32(18.2-28.5)	21.28(18.4-25.9)	0.798
Venous	22.85(19.9-26.5)	23.22(19-29.1)	0.535

Migraine relief in patients with and without aura was also compared. No significant difference was found in the reduction of the frequency and intensity of the migraine in these two cohorts post-NBO (all *p* > 0.05).

### Blood analysis after NBO

Data on arterial and venous blood gases were also recorded and compared before and after NBO treatment. There was no significant difference in arterial oxygen partial pressure (PaO_2_) before and after NBO treatment (*p* = 0.492). These findings suggested that 8 L/min of oxygen inhalation for 1 h is safe and effective. In this study, a remarkable increase in arterial PaO_2_ was observed within 2 h post-NBO (the PaO_2_ at baseline vs. 2nd -hour post-NBO, *p* < 0.01) (Table 2).

**Fig. 2:**
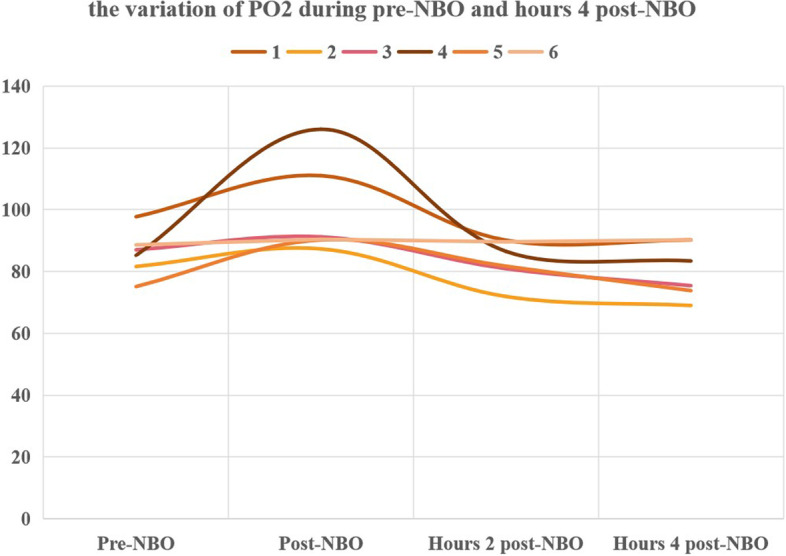
The variation of PaO_2_ during before NBO and 4 h after NBO are showed, there is significant increase after NBO, and the value of PaO_2_ fall down gradually during the 4 h after NBO

Additionally, dynamic arterial blood gases post-NBO were monitored in 6 patients to observe the variation of the PaO_2_ post-NBO (Fig. 2). It’s noteworthy to note that the elevated PaO_2_ progressively returned to the baseline at about the 4th hour after treatment with NBO.

#### EEG manifestation

**Fig. 3:**
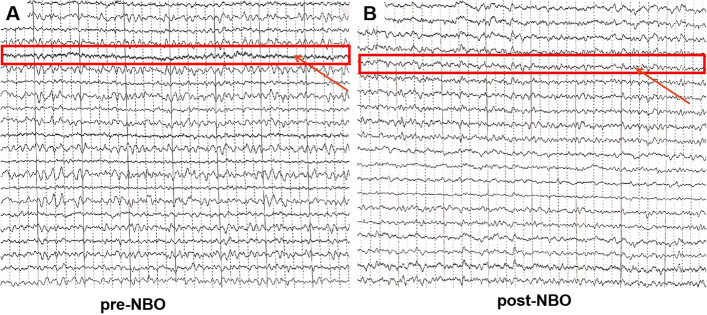
Difference of brain activity between before and after NBO. **A **slow waves are found on the temporal cortex of patients with migraine before undergoing NBO. **B **the brain activity was normal after undergoing NBO

Five patients underwent EEG scanning at 8 and 10 AM in a warm and quiet room, before and 1 h after NBO, respectively. 4(80%) patients who showed abnormal slow waves in bilateral hemispheres prior to NBO disappeared post-NBO (Fig. 3).

## Discussion

To date, this is the first study to assess the efficacy of NBO in relieving PFO-migraines. The results show that NBO may attenuate the frequency and intensity of headaches in patients with PFO and migraines. Furthermore, 1-hour of NBO was safe, as it raised PaO_2_ but did not cause fluctuation of other values in blood gas measured simultaneously.

According to analysis of the dynamic blood gas and EEG results, 1-hour of NBO could relieve migraine and diminish abnormal slow waves in EEG. NBO may be a promising adjuvant treatment for attenuating the frequency and intensity of PFO- migraines.

### NBO may inhibit PFO-migraine

Baseline PaO_2_ analysis results indicate that the level of PaO_2_ in patients with PFO-migraine was significantly lower than that in healthy volunteers. This finding provides real-world evidence to support the relationship between PFOs and migraine.

One of the main hypotheses is that PFO-induced RLS caused venous blood to mix with arterial blood, resulting in lower blood oxygen content being delivered to the brain [9, 10], and therefore hypoxemia in the brain [13]. Surprisingly, several clinical investigations have shown that hypoxia may cause migraines and auras [14]. Despite the fact that numerous hypoxia pathways have been hypothesized, the putative hypoxic mechanisms in migraines remains unclear.

Current literature suggests that hypoxia produces NO, calcitonin gene-related peptide(CGRP), adenosine, hypoxia-inducible factor 1 (HIF-1), vascular endothelial growth factor(VEGF), and other regulatory mediators, which results in meningeal vasodilation, increased blood flow, and angiogenesis, leading to the phenotypic presentation of migraine [15, 16]. HIF, in particular, seems to be an important regulator since it not only generates VEGF, but also causes disruption of the blood-brain barrier (BBB) and forms reactive oxygen species (ROS) through oxidative stress [17].

**Fig. 4:**
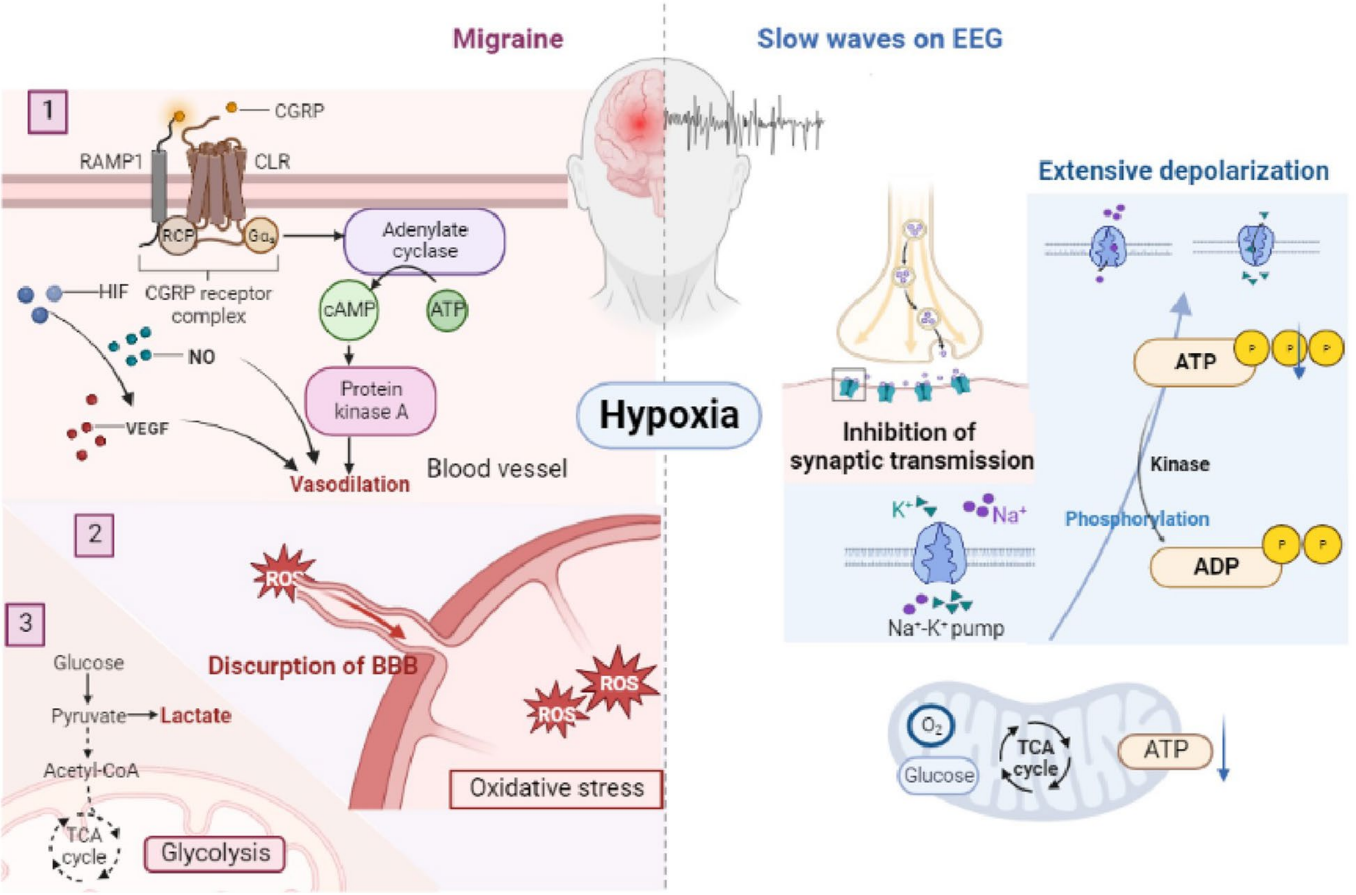
Hypothesis of how hypoxemia causes migraine and slow waves on EEG

In addition, when the brain is exposed to hypoxia, it is able to generate energy anaerobically through glycolysis. The anaerobic generation of energy results in the production of lactate, which is a potential trigger of migraines. (Fig. 4) [18]. On the basis of these mechanisms, it is reasonable to postulate that migraines and PFO may be associated with hypoxemia in the tissue of the brain. In addition, our discovery brought attention to the connection between cortical spreading depression (CSD) and PFO [10]. In the 1940s, Leo, A. A. et al. had previously identified that slowly moving waves traveling over the surface of the brain indicates CSD [19]. Surprisingly, CSD was shown to be resistant to the dilatation of vessels and may be considered as the pathological basis for migraine [10]. More specifically, Nozari A et al. indicated that microemboli and hypoxia could trigger CSD [20, 21], which may explain the connection between PFO-migraine and CSD [22].

In spite of this, patients continued to experience their migraines inspite of certain clinical research predicting that nasal oxygen would somewhat lower the intensity of migraines [4, 23]. Current literature suggests that NBO is a safe and convenient treatment for improving short-term symptoms and long-term clinical outcomes of hypo-perfusion and hypoxia in brain tissue by increasing oxygen content [12]. Particularly, there is real-world evidence for safety and efficacy profile of NBO for chronic cerebral circulation insufficiency and acute intracerebral hemorrhage-mediated brain damage in randomized controlled trials [24]. Moreover, there are some reports on the benefit of oxygen inhalation as an effective acute medical treatment in cluster headache, migraine, and other headache disorders [25]. Furthermore, our findings show that NBO may greatly decrease the severity of migraines during the ictal phase. We hypothesize that the mechanism may involve NBO improving the concentration of PaO_2_ in blood circulation.

Paradoxical embolisms and the accumulation of vasoactive substances such as serotonin and CGRP are two further pathophysiological connections between PFO and migraine that are widely accepted [26]. Furthermore, Farid Khasiyev et al. demonstrated that these vasoactive substances could via escaping from the pulmonary circulation through PFO and subsequently into cerebrovascular circulation then trigger reduction of cerebral vasomotor reactivity (CVMR), moreover, a decrease in CVMR would slow the removal of the microemboli, which would further compromise CVMR [10]. Therefore, several reports suggested that decreasing CVMR may be a key mediator mechanism for some migraine triggers in PFO patients [27]. CSD has also been discovered to be a potential contributing factor to CVMR impairment [28]. CVMR may fill some of the gaps in our knowledge of the pathophysiology of PFO and migraine given that several studies regarding PFO have shown that these paradoxical embolisms of PFO may trigger CSD in animal models and directly cause migraine [10]. As a result, numerous physicians tried to develop a novel treatment by increasing the cerebral vascular activity of migraine patients, however several medications failed to effectively regulate cerebral vascular activity in these individuals [26]. It’s intriguing to note that NBO is reported to have the capacity to regulate the vasodilator effect on cerebral vascular circulation [28]. Therefore, NBO may not only reduce cerebral hypoxia in individuals with migraine and PFO but also demonstrate its effects through vasoconstriction. Furthermore, our data, which shows an association between migraine and PFO, indicates that lower oxygen concentration in brain tissue and VMR impairment may be the key pathogenic links between migraine and PFO.

However, in this study there was no reduction in PCO_2_ and TCO_2_ after one-hour of NBO. As a result, we propose that 1-hour of NBO is insufficient to produce a change in CO_2_ concentration; hence, the approach of standardized 1-hour NBO may be safe and viable.

### NBO may abrogate abnormality of brain activity

In this study, the EEG of PFO-migraine patients during the ictal phase revealed a slowly propagated wave of brain activity. Previous research also revealed that hypoxia is a major cause of slow-wave components in EEG [29]. The widely accepted theory is that brain function is influenced by oxygen supply, as glucose and oxygen are the two most important substrates for oxidative metabolism in the brain. Under hypoxic conditions, mitochondria are unable to produce enough ATP to maintain activity of ion pumps in the brain [13]. This results in a series of changes to transmembrane electrochemical gradients, such as rapid and widespread membrane depolarization, which may lead to widespread depression of synaptic transmission and slowing of electric activity [2] (Fig. 4). Alterations in EEGs of hypoxic patients may also be explained by increased blood flow to the brain and slowed brain functional activity that compensates for the brain’s inability to produce enough oxygen under hypoxic conditions [30]. Since hypoxemia contributes to the pathophysiology of PFO-migraine, we hypothesized that it was the cause of the abnormal EEG activity in PFO-migraine patients. Particularly, Eser Basak Sevgi et al. found that paradoxical embolism could cause transient hypoxia and then migraine, which is unique to patients with PFO-migraine [28]. In patients with migraines and no PFOs there was no abnormal electrical activity detected. Therefore, the presence of slow waves may be a typical characteristic of patients with PFO-migraines in our study [18]. In addition, Hisaki Ozaki et al. suggested that hypoxia-induced slowing waves on EEG could be reversed with NBO [26].

However, it should be noted, that although EEG is useful as a simple and convenient approach for monitoring, it cannot offer quantitative assessments of changes in brain activity. Thus, all patients had a four-hour EEG while calm and in a war, quiet room to minimize severe fluctuations from external stimuli owed to varied vigilance states and autonomic nervous system activation. Furthermore, rather than through spectral power analysis, EEG data was examined visually in this study. As such, these biases may result in false-positive results; therefore, larger-scale clinical trials are required to eliminate bias [31].

### The advantage of NBO

Another clinically significant result was that NBO-mediated intolerance and adverse effects were rare and if they occurred, mild. According to the findings of this investigation, if a patient were to experience intolerance or an adverse effect, these were all resolved by halting NBO therapy. Furthermore, NBO provides a safer risk profile compared to painkillers in treating migraines. Dangerous side effects of pain killers include hepatic and renal impairment. In addition, it is well known that prolonged use of painkillers may lead to intolerance. As a result, NBO is preferable as a treatment compared to both painkillers and surgical procedure. During the duration of NBO treatment, we monitored the change of pH, oxygen, and carbon dioxide levels; we observed no acid-base imbalance or oxygen poisoning. Furthermore, NBO therapy can be derived either at home or in the hospital via a mask connected to an oxygen cylinder and a home oxygen concentrator [32]. In this study, we also discovered that the increased PaO_2_ from 1-hour of NBO treatment could last for approximately 4 h before gradually declining. As a result, it is reasonable to hypothesize that 3 rounds of 1-hour NBO inhalation daily may maintain a continuously high levels of PaO_2_, which may help prevent PFO-migraine attacks. Further studies are required to support these claims.

### The association between migraine and the size of PFO

Migraines are widely documented as a prevalent consequence of RLS through the PFO tunnel. Schwerzmann M et al. found that a moderate or large RLS through a PFO resulted in a greater incidence of migraine [29]. Based on this data, current guidelines only recommend that PFO-migraine patients undergo closure if they have a medium or large RLS detected by TEE or bubble-TCD. Therefore, PFO-migraine patients with small RLS are unable to undergo PFO closure despite having regular migraines [25]. However, our data did not show a significant association between the size of the RLS and the incidence of migraines. Patients with small RLS were just as likely to have the same number of migraines as patients with medium or large RLS. This is inconsistent with the results noted previously, and our findings in this study suggest that small RLS may be worth examining and treating. Our results, however, may be limited due to the small sample size of our study. As such, studies with larger sample-sizes should be carried out to investigate the relationship between RLS and incidence of migraines. In spite of this, we still recommend that PFO-migraine patients undergo treatment regardless of the quantity of RLS.

## Limitations

Our study has several limitations. The small sample size and resulting bias from the lack of a blinding method may cause false-positive results about the reduction in intensity of migraine. Secondly, there was unavoidable bias from patients using the subjective VAS scale to assess their migraines. Furthermore, in this study, the EEG signal was evaluated visually rather than through spectral power analysis or other methods. This may result in a biases interpreting the electric activity PFO-migraine patients.

## Conclusion

Patients with PFO–migraine may derive benefit from NBO treatment. PFOs result in arterial hypoxemia due to mixing of venous blood, which ultimately results in brain hypoxia and migraines. This series of events may be the key pathologic link explaining how PFOs lead to migraines. NBO treatment may attenuate the headaches from migraines by correcting the hypoxemia.

## Data Availability

All data generated or analysed during this study are included in this published article.

## Abbreviations

migraine-PFO: Patent foramen ovale with migraine
PFO: Patent foramen ovale
NBO: Normobaric oxygenation
RLS: Right-to-left shunts
PaO_2_: Oxygen partial pressure
PaCO_2_: Carbon dioxide partial pressure of carbon dioxide
SpO: Oxygen saturation
TCO: Total carbon dioxide
bubble-TCD: Contrast-enhanced transcranial Doppler
TEE: Transoesophageal echocardiography
EEG: Electroencephalo-graph
MRI: Magnetic resonance imaging
CSD: Cortical spreading depression
WHL: Whiter matter lesions
CVMR: Cerebral vasomotor reactivity
